# Evaluating the Accuracy of ChatGPT in the Japanese Board-Certified Physiatrist Examination

**DOI:** 10.7759/cureus.76214

**Published:** 2024-12-22

**Authors:** Yuki Kato, Kenta Ushida, Ryo Momosaki

**Affiliations:** 1 Department of Rehabilitation, Saiseikai Meiwa Hospital, Meiwa, JPN; 2 Department of Rehabilitation Medicine, Mie University Graduate School of Medicine, Tsu, JPN; 3 Department of Rehabilitation, Mie University Hospital, Tsu, JPN

**Keywords:** artificial intelligence, generative ai, machine learning, medical education, rehabilitation medicine

## Abstract

Background

Generative artificial intelligence (AI), such as Chat Generative Pre-trained Transformer (ChatGPT), has shown potential in various medical applications, including answering licensing examination questions. However, its performance in rehabilitation medicine remains underexplored. This study aimed to evaluate the accuracy of ChatGPT4o in answering questions from the Japanese Board-Certified Physiatrist Examination and assess its potential as an educational and clinical support tool.

Methods

This study assessed the performance of ChatGPT4o on questions from the 2021-2023 Japanese Board-Certified Physiatrist Examinations. Questions were categorized into text- and image-based types and correct response rates were calculated. Errors were classified into informational, logical, or statistical.

Results

ChatGPT4o achieved correct response rates of 79.1% in 2021, 80.0% in 2022, and 86.3% in 2023, with an overall accuracy of 81.8%. The AI performed better on text-based (83.0%) than on image-based (70.0%) questions. Most errors (92.8%) were related to information.

Conclusions

ChatGPT4o demonstrated high accuracy in the Japanese Board-Certified Physiatrist Examination, particularly for text-based questions, demonstrating its potential as an educational tool. However, limitations in image interpretation and specialized topics indicate the need for further improvements for clinical application.

## Introduction

In recent years, generative artificial intelligence (AI) has rapidly evolved and become increasingly adept at creating new data by leveraging existing datasets. Initially, generative AI focused on producing images and audio [1]. However, the introduction of the transformer architecture has substantially advanced natural language processing (NLP), greatly enhancing the ability to generate coherent text. This architecture involves pretraining the transformer model on large datasets, followed by fine-tuning for specific tasks, which enables generative AI to produce more natural and contextually consistent text. Its utility has been demonstrated across various applications [2]. A prominent example is the Chat Generative Pre-trained Transformer (ChatGPT), an NLP model released by OpenAI in 2022, which has seen rapid adoption across numerous fields [3].

In medicine, ChatGPT has garnered attention for its potential applications. It is evaluated as a tool for supporting diagnostic and treatment decisions and generating medical documentation [4,5]. Notably, ChatGPT has shown strong performance in the United States Medical Licensing Examination (USMLE), where it has been used to answer examination questions [6,7]. For instance, Kung et al. (2023) reported that ChatGPT3 achieved a correct response rate of 50%-60% on USMLE questions, approaching the passing threshold of 60%. Although results varied depending on the input, Yaneva et al. found that ChatGPT3.5 generally exceeded the passing standard.

ChatGPT is a generative AI developed through extensive pretraining on large volumes of text data, followed by fine-tuning for specific tasks. During pretraining, the language model learns to predict the next word using a broad range of data from the Internet [8]. The performance of ChatGPT varies depending on the linguistic characteristics and training data volume [9].

ChatGPT, particularly in its application to medical licensing examinations in Japanese, has demonstrated varying proficiency levels depending on the specific field of medicine and the complexity of questions. Previous studies have examined the ability of ChatGPT to answer questions from Japanese medical licensing examinations for physicians, pharmacists, and physical therapists [10-12]. For example, Kasai et al. evaluated ChatGPT4, which was the latest model at the time, on the national medical licensing examination for physicians and found that it exceeded the passing threshold. Similarly, studies on examinations for pharmacists and physical therapists showed that ChatGPT4 performed above the passing standard, highlighting its capability in these domains. However, in a specialized otolaryngology examination conducted in Japanese, ChatGPT4 achieved only a 45% correct response rate, indicating lower performance than other reports [13]. Despite these findings, no studies have evaluated the performance of ChatGPT on specialized examinations in rehabilitation medicine, leaving a gap in our understanding of its capabilities in this area.

Interest in rehabilitation extends beyond multidisciplinary teams to include patients and their families [14]. Given the widespread utilization of ChatGPT as an information retrieval tool, it will likely be employed to obtain rehabilitation-related information [15]. Thus, evaluating whether ChatGPT can provide accurate and reliable knowledge in this area is crucial. If proven reliable, ChatGPT could be a valuable tool for expert education and clinical practice support.

This study aimed to assess the accuracy with which ChatGPT4o, the latest version of ChatGPT, can answer questions from the Japanese Board-Certified Physiatrist Examination. Based on the findings, this study sought to provide guidelines on utilizing generative AI to disseminate specialized knowledge in rehabilitation medicine and as an educational and clinical support tool. For this study, questions from the 2021-2023 Japanese Board-Certified Physiatrist Examinations were used; ChatGPT4o answered them, its accuracy was analyzed, and any errors were classified.

## Materials and methods

Japanese Board-Certified Physiatrist Examination

This study utilized data from the Japanese Board-Certified Physiatrist Examination from the past three years (2021-2023), obtained from the members-only section of the Japanese Association of Rehabilitation Medicine website. All data written in Japanese included question texts, answer options, and correct answers. The examination comprised 150 questions, which included both text- and image-based questions. All questions are formatted with multiple choices, requiring the selection of either correct or incorrect options. Any questions identified as inappropriate were excluded.

The Japanese Board-Certified Physiatrist Examination includes basic medical knowledge related to rehabilitation medicine, treatment of specific diseases, and relevant laws and social systems. However, these questions are not classified. For this study, the questions were divided into two categories based on whether they included images. To analyze the nature of the questions, we classified them into four qualitative categories:(1) Concept-based questions, which assess the understanding of specific concepts; (2) Critical thinking questions, which require judgment and reasoning; (3) Comparative questions, which involve selecting the most appropriate option through comparison; and (4) Factual questions, which evaluate knowledge of clearly defined facts.

ChatGPT

In this study, ChatGPT4o, the latest version of ChatGPT provided by OpenAI, was used and evaluated in August 2024. Released in May 2024, ChatGPT4o offers improved speed and efficiency over previous models and can process nontext data, such as audio, images, and videos [16].

Answer generation and evaluation

The questions from each year’s examination were inputted into ChatGPT4o, and plain-text answers and explanations were obtained. The test questions were input in Japanese, and the responses and explanations were all in Japanese. For questions that included images, both text and images were keyed simultaneously. The correct response rate for each year and category was calculated, and the text-based questions and image-based questions were compared using Fisher's exact test. Incorrect responses were classified into three categories based on the explanations generated by ChatGPT4o and a previous study [17]: 1) Information error: Incorrect answers caused by inaccurate information within the response, for instance, if ChatGPT4o incorrectly answers a question about disability certificates because it provides inaccurate information about the legal system; 2) Logical error: Incorrect answers despite arriving at an apparently correct conclusion, for example, if ChatGPT4o offers two correct options for a question that requires only one correct answer; 3) Statistical error: Incorrect answers resulting from calculation errors or similar issues.

## Results

Inappropriate questions from the Japanese Board-Certified Physiatrist Examinations from 2021 to 2023 were excluded. The 2021, 2022, and 2023 examinations included 149, 150, and 147 questions, respectively. In the 2021 examination, 135 and 14 were text- and image-based questions, respectively. The 2022 examination included 137 text- and 13 image-based questions. The 2023 examination had 133 text- and 14 image-based questions. In total, across the three years, there were 406 text- and 40 image-based questions.

Correct response rates

Table 1 shows the correct response rates for each year. ChatGPT4o achieved a correct response rate of 118 (79.1%) in 2021, 120 (80.0%) in 2022, and 127 (86.3%) in 2023. Overall, it correctly answered 365 out of 446 questions, resulting in an overall accuracy rate of 81.8%. When analyzing the three-year period, the correct response rates were 337 (83.0%) and 28 (70.0%) for text- and image-based questions, showing a higher performance for text-based questions, but there was no significant difference overall. In comparisons between each year, only the 2023 examination showed a significant difference. Table 2 shows the correct response rate for each qualitative classification. ChatGPT-4o achieved 170 (88.1%) correct answers for factual questions, 20 (83.3%) for concept-based questions, 85 (77.3%) for comparative questions, and 90 (75.6%) for critical thinking questions. The correct response rate for concept questions and factual questions was higher than the overall rate.

**Table 1:** Number of questions and correct response rates Data presented in the form of n (%). p-value > 0.05 (non-significant). P-value using Fisher's exact test.

	Total number of questions	Text-based questions	Image-based questions		Overall correct response rate (%)	Text-based Correct (%)	Image-based Correct (%)	p-value
2021	149	135	14		118 (79.1)	108 (80.0)	10 (71.4)	0.491
2022	150	137	13		120 (80.0)	111 (81.0)	9 (69.2)	0.294
2023	147	133	14		127 (86.3)	118 (88.7)	9 (57.1)	0.0251
Total	446	406	40		365 (81.8)	337 (83.0)	28 (70)	0.0523

**Table 2:** Classification of qualitative categories Data presented in the form of n (%).

	Concept-based question	Critical thinking question	Comparative question	Factual question	Total
Total number of questions	24	119	110	193	446
Correct response rate (%)	20 (83.3)	90 (75.6)	85 (77.3)	170 (88.1)	365 (81.8)

Error analysis

Table 3 categorizes the types of incorrect answers. Among the text-based questions, information errors accounted for 90 (95.7%). The remaining four (4.3%) were logical errors. For image-based questions, information errors also predominated, accounting for 10 (76.9%); however, they have more logical errors than text-based questions. No statistical errors were found in either type of question.

**Table 3:** Classification of incorrect answers Data presented in the form of n (%).

	Text-based questions	Image-based questions	Total
Total errors	69	12	81
Information errors (%)	64 (92.8)	9 (75.0)	73 (90.1)
Logical errors (%)	5 (7.2)	3 (25.0)	8 (9.9)
Statistical errors (%)	0 (0.0)	0 (0.0)	0 (0.0)

Exploratory analysis

Further exploratory analysis revealed that the incorrectly answered questions spanned a wide range of topics. These included questions related to systems such as activities of daily living assessments (e.g., functional independence measure and Barthel index), assessments for individuals with higher brain dysfunction, disability certificates, and community-based care wards.

## Discussion

This study evaluated the performance of ChatGPT4o in the Japanese Board-Certified Physiatrist Examination, which demonstrated a high correct response rate of >80%. ChatGPT4o demonstrated better performance on text- than on image-based questions, although there was a lack of statistical significance. This may have been due to the small number of image-based questions. The errors observed spanned various fields, and information errors were the most common, particularly in text-based questions. However, the incidence of logical errors was higher in image-based questions. In the qualitative classification of questions, purely factual- and knowledge-based questions were more prevalent and showed higher correct response rates. This may be because questions requiring critical thinking or comparison tend to demand the application of knowledge, making them more challenging for ChatGPT.

These findings are generally consistent with the results of previous studies that have shown the strong performance of ChatGPT in the medical field. In one exception, a study on the Japanese otolaryngology specialist examination reported a correct answer rate of 45.42% using the Japanese version of ChatGPT4, which was lower than the rate observed in this study [13]. The difference in performance could be attributed to the ChatGPT version used or the timing of the evaluation. However, as neither study disclosed the exact passing standard, direct comparison between the studies is difficult.

Most incorrect responses were attributed to information errors, with fewer logical errors and the absence of statistical errors. The higher incidence of logical errors in image-based questions proposes that although ChatGPT4o has capabilities for image analysis, its proficiency in this area may still be less developed compared with its language processing abilities. The lack of statistical errors may be due to the nature of the Japanese Board-Certified Physiatrist Examination, which does not include many questions that require calculations. In addition, the examination is designed by a committee of supervising physicians from the Japanese Association of Rehabilitation Medicine, which may contribute to the logical clarity of the questions.

Moreover, ChatGPT4o underperformed in specialized fields such as evaluation scales. This may be due to the limited training data in Japanese that includes specific legal information or specialized medical knowledge, which contrasts with the broader availability of medical knowledge in English [18]. In addition, legal systems and evaluation criteria can change over time, potentially leading to outdated information in the training data [19].

This study has several limitations. First, the variability in responses generated by ChatGPT makes it difficult to ensure reproducibility. To mitigate this, the ChatGPT version was standardized, and evaluation was conducted within a specific time frame. Second, the classification of test questions and incorrect answers relied on the subjective judgment of the researchers. Although we referenced previous studies to enhance the validity of our classifications, information errors dominated the errors identified. Furthermore, the response generation capabilities of ChatGPT in this study were limited to multiple-choice questions within the domain of Japanese rehabilitation medicine. It is important to note that multiple-choice and essay-based questions assess different cognitive skills, and AI may perform less effectively on essay-based questions that require complex reasoning [20,21]. Moreover, its ability to generate accurate responses may differ across other examination fields.

Despite these limitations, ChatGPT4o could be a valuable tool in rehabilitation medicine education. Given its high accuracy with simple text inputs, it may serve as a reliable educational support tool when used with appropriate prompts and more training data. Moreover, if ChatGPT can provide accurate answers to patient and family inquiries, it could enhance awareness and engagement in rehabilitation practices. Studies have reported using ChatGPT to generate test questions for medical education, and it may be used in the future to support the creation of the Japanese Board-Certified Physiatrist Examination. In addition to generating potential questions, ChatGPT could be useful in assessing question difficulty by response analysis.

However, the potential for ChatGPT to generate incorrect answers and its inconsistent performance present challenges for its use as a clinical support tool. The high error rates in questions related to evaluation scales in actual clinical practice highlight this concern. OpenAI has expressed caution regarding AI use in critical clinical decision-making, highlighting the need for ongoing performance verification [22].

## Conclusions

ChatGPT4o demonstrated strong performance in the Japanese Board-Certified Physiatrist Examination, with an overall accuracy rate of >80%. ChatGPT4o showed high performance in text-based questions, although the difference was not statistically significant. ChatGPT4o tended to make errors when it was unable to use appropriate knowledge and information, and it had difficulty with questions that changed over time, such as those related to systems.

This finding proposes its potential as a valuable tool in rehabilitation medicine, particularly in educational settings. In addition, ChatGPT4o may be even more effective if it is given appropriate information data to learn in advance. However, limitations in image interpretation and specialized areas require further refinement for clinical application. Future studies should focus on improving its capabilities in these areas to fully leverage its potential in both education and patient care.
